# Patient and Provider Perspectives on Enrollment in Precision Oncology Research: Qualitative Ethical Analysis

**DOI:** 10.2196/35033

**Published:** 2022-05-03

**Authors:** Kayte Spector-Bagdady, Madison Kent, Chris D Krenz, Collin Brummel, Paul L Swiecicki, J Chad Brenner, Andrew G Shuman

**Affiliations:** 1 Center for Bioethics and Social Sciences in Medicine University of Michigan Medical School Ann Arbor, MI United States; 2 Department of Obstetrics and Gynecology University of Michigan Medical School Ann Arbor, MI United States; 3 Michigan Otolaryngology and Translational Oncology Laboratory University of Michigan Medical School Ann Arbor, MI United States; 4 Division of Hematology-Oncology Department of Medicine University of Michigan Medical School Ann Arbor, MI United States; 5 Department of Otolaryngology-Head and Neck Surgery University of Michigan Medical School Ann Arbor, MI United States

**Keywords:** oncology research platform, precision oncology, head and neck oncology, academic cancer center, semistructured interview, patient-provider dyads, oncology, interview, ethical analysis, patient, provider

## Abstract

**Background:**

The genomic frontier continues to revolutionize the practice of oncology. Advances in cancer biology from tumorigenesis to treatment resistance are driven by the molecular underpinnings of malignancy. The framing of precision oncology as both a clinical and research tool is constantly evolving and directly influences conversations between oncologists and their patients. Prior research has shown that patient-participants often have unmet or unrealistic expectations regarding the clinical utility of oncology research and genomic sequencing. This indicates the need for more in-depth investigation of how and why patients choose to participate in such research.

**Objective:**

This study presents a qualitative ethical analysis to better understand patient and provider perspectives on enrollment in precision oncology research.

**Methods:**

Paired semistructured interviews were conducted with patient-participants enrolled in a prospective head and neck precision oncology research platform, along with their oncology providers, at a National Cancer Institute–designated academic cancer center.

**Results:**

There were three major themes that emerged from the analysis. (1) There are distinct and unique challenges with informed consent to precision medicine, chiefly involving the ability of both patient-participants and providers to effectively understand the science underlying the research. (2) The unique benefits of precision medicine enrollment are of paramount importance to patients considering enrollment. (3) Patient-participants have little concern for the risks of research enrollment, particularly in the context of a low-burden protocol.

**Conclusions:**

Patient-participants and their providers offer complementary and nuanced perspectives on their motivation to engage in precision oncology research. This reflects both the inherent promise and enthusiasm within the field, as well as the limitations and challenges of ensuring that both patient-participants and clinicians understand the complexities of the science involved.

## Introduction

The genomic frontier continues to revolutionize the practice of oncology. Advances in cancer biology from tumorigenesis to treatment resistance are driven by the molecular underpinnings of malignancy, and the framing of precision oncology as both a clinical and research tool is constantly evolving. Introspection is warranted to examine how conversations between oncologists and their patients may be affected.

Studies have assessed the motivations of research participants enrolling in genome sequencing research, such as the HealthSeq [1] and ClinSeq [2] projects, and reflected the tension between the risk and potential reward that these platforms offer. Additional studies have explored the perspectives of patient-participants enrolled in precision oncology studies, many of whom reported unfilled expectations [3]. These patient-participants also reported a higher level of perceived utility of the study at the time of enrollment than after enrollment. Specifically, their expectations that participation in a genome sequencing study would affect future health and medication decisions were not frequently met [4].

These studies all indicate the need for more nuanced questions and perspectives. As one study states, “Further evaluation of whether and how family members and close contacts were involved in the patient’s decision to pursue or decline sequencing, and any discussion with family members and friends preceding sequencing, may help to elucidate how these dynamics affect decision-making” [5]. A key component when asking these questions is to address the unique concerns in this field of research. For example, precision oncology has a more established clinical utility in certain cancers than others. Moreover, the role of germline mutations is de-emphasized in many cancers, which may confuse how patients consider the issues of heritability and familial risk. In addition, cancer stage, prognosis, and recurrence will all invariably impact how patients, many of whom are affected by cancers considered to be terminal, will consider the prospect of using “cutting edge science” to save their lives. This is particularly true when most precision oncology platforms to date have had, at best, modest impact on survival outcomes.

Our aim is to better understand patient and provider perspectives related to the decision to enroll in a low-burden precision oncology protocol. In this study, we employed a qualitative embedded ethics protocol involving semistructured interviews of both adult patients with head and neck cancer enrolled in precision medicine research and their clinicians. This study was nested within a prospective precision oncology study at one institution, a National Cancer Institute–designated academic cancer center. Two other articles have been derived from the interview data set, one focused on patient and provider perspectives on enrolling in head and neck cancer research [6] and the other on commercialization of cancer genomic data [7]. Herein, we focus specifically on patient and provider perspectives on enrollment in precision oncology itself.

## Methods

### Overarching Study Design

This inquiry ran alongside the overarching study, “Developing Precision Medicine Protocols for Head and Neck Cancer MiOtoSeq (Michigan Otolaryngology and Translational Oncology Sequencing Center),” an institutional review board–approved precision medicine study in the Michigan Medicine Department of Otolaryngology-Head and Neck Surgery [8]. Patient-participants enrolled in MiOtoSeq were adults with biopsy-confirmed cancer of the head and neck who were counseled and consented to participate in upfront, targeted genomic research sequencing of their tumors and germline tissues. In conjunction with the MiOtoSeq study, we embedded this qualitative ethics protocol to better understand and compare perspectives on their involvement in precision oncology research. Specifically, we were interested in the motivations of patients and providers to enroll in the research.

### Interviews

A subset of the MiOtoSeq patient-participants were purposively sampled for interviews based on demographic and clinical factors to ensure a diverse variety of experiences. All patients participated in a 1-hour interview conducted by researchers trained in semistructured interviewing techniques [9]. All interviews were conducted in 2018.

The interviews were audiorecorded, transcribed by a third-party service, and deidentified. All interview files were stored on an institutionally supported secure storage platform. In these interviews, participating patients and clinicians were asked a variety of questions related to the goals of precision medicine research, the risks and benefits as they perceived them, and their experience with the MiOtoSeq enrollment and consent process.

This analysis includes responses from a total of 20 interviews from 10 patients and 8 clinicians. In the cases of 2 physicians, each had treated 2 patients and we conducted 2 separate interviews with the physicians to focus on each patient. Patient-participants were recruited until thematic saturation was achieved [10] and then their physician was recruited for comparison purposes. One of the clinicians is an author of this analysis, and his interview responses were excluded from quotation. Once the interviews were underway, team members (KSB and MK) iteratively developed the codebook [9]. Transcripts were inductively and deductively double-coded (by MK and CK) and discordances were reconciled (KSB). Please refer to our previous publication for more detail regarding these methods [6]. For the purposes of this article, gender pronouns for clinicians and patient-participants were randomly selected for additional privacy.

### Ethics Approval and Consent to Participate

Approval was obtained from the ethics committee of the University of Michigan (HUM00085888). The procedures used in this study adhere to the tenets of the Declaration of Helsinki. Informed consent was obtained from all individual participants included in the study.

## Results

### Theme 1: Challenges With Informed Consent to Precision Medicine

Many patient-participants stated that their background knowledge of genetics came from media or television. For example, several patient-participants cited the movie Jurassic Park, coverage of “test tube babies,” or the Discovery Channel as their main source of genetic information. As one patient-participant put it, their awareness began “when Francis (or was it Crick?) first started the Human Genome Project” (Patient [P] 05). As one clinician aptly joked, “I think most patients don’t understand [genetics], because I barely do in a lot of ways” (Clinician [C] 10).

Many clinicians were concerned that the patients’ lack of understanding of genetics, and research in general, might lead to conflations between clinical care and enrollment in a precision medicine protocol. For patients without a strong grasp of the basics of genetics, the nuanced potential benefit of precision oncology—where clinical care and research may be blurred—was complex to understand. For example, the doctor of one of the patient-participants who said he had learned about genetics from Jurassic Park admitted that although he explained to the patient that this was not a therapeutic trial, “…maybe he didn’t get that. I don’t know [laughs]” (C02). Another doctor added, “I don’t know if [my patient] actually understood, because patients express understanding of almost *everything* I say…” (CO3). Other clinicians seemed reassured that patients at least understood that the research would not change their clinical care or help them directly. However, despite one clinician stating that he thinks “the personal reward for any individual patient is very low” (C11), his patient stated that her expectation from participating in the study was that it “might save my life” (P11).

Other clinicians emphasized the inherent vulnerability of patients in a clinical oncology visit and how that might compound confusion or inadvertent exploitation. Of note, although the clinicians were MiOtoseq coinvestigators, consent for enrollment into the study was obtained by a dedicated study coordinator. One clinician described her realization that “most patients don’t understand genetic sequencing and simply sign something because we give [it to] them in a very vulnerable situation” (C07). She went on to describe asking patients to enroll in research during a clinical care visit as “really not an informed consent process.” Another clinician agreed that his patients were “more worried about not passing away from [the cancer] as opposed to having their sequencing done” (C10). As a patient affirmed, “In the whirlwind of things…I really didn’t think about [enrolling in research] too much…I just consented” (P11). However, a different patient-participant described the benefit of learning about precision medicine in the clinical context: “Wow, you know, I’d like to know more about myself…and my genetic makeup and kind of what went wrong…” (P08).

### Theme 2: Unique Benefits of Precision Medicine Enrollment

Many patient-participants were excited about the promise of precision medicine research specifically, referring to current cancer treatment options as “archaic.” They described precision medicine as “the future,” and several expressed hope for finding a cure for cancer.

I think that we have no idea of what we’re doing right now. We’re dabbling a foot in the pool, but once we get all the way into that pool, I think we’re going to have some serious answers.

P07

Patient-participants were less clear about potential benefits to themselves in enrolling in precision medicine research. Although the majority noted that they realized the research was not primarily for their own benefit, many held out hope for the “teeny, teeny, teeny, teeny possibility [that] it could help me” (P07). Several patient-participants specifically described hoping that the research could help them if their cancer came back in the future. Clinicians appeared generally aware of their patients’ aspirations to have their cancer cured, which one described as a “common coping strategy” (C07). Although, as one clinician said, he explains to patients that the research could not possibly affect their clinical course, “when it takes 14 months to get the sequencing back!” (C02).

More uniquely related to a precision medicine protocol than other types of clinical research, many patient-participants also described that research participation might help their blood relatives in the future and protect them from “what is inside me that came from my ancestors…” (P04). Almost all spoke about protecting their family and children through research enrollment, with one patient-participant stating that they “would do anything to make sure they [their children] don’t go through this” (P08). Another described this altruistic legacy as “a way for me watching out for my family later on when I’m gone” (P07). Another added: “I would hope that this could help, you know, my family first and then out into other people” (P09). Notably, some of these themes might relate to other novel cancer research platforms and are not necessarily specific to precision oncology itself.

### Theme 3: Risks of Research Enrollment

Although patient-participants overwhelmingly spoke of hope and the potential benefits of precision medicine research, the majority of those who spoke of risks only brought them up to dismiss them. Many discussed how enrolling in a precision medicine protocol had no additional risk or burden to themselves and did not involve much effort or downside: “If there’s something that really doesn’t cause you any…discomfort, really takes up very little of your time, if down the road 30 or 40 years from now, that could really affect peoples’ lives, you know, why wouldn’t you want to do that?” (P09). One patient-participant also discussed the convenience of being able to complete everything in the same visit; he said that if the trial required extra visits, he probably would not have enrolled.

If patient-participants or their clinicians mentioned specific risks that concerned them, the most common was finding out information that the patients might not want to know. One patient-participant described these potential secondary findings as both “a shield and a sword” (P05). She added, “I can’t see that ignorance could possibly benefit you…other than a bit of bliss I suppose.” Another patient-participant dismissed the risk of finding out unwanted information this way: “Life has twists and turns. We don’t have a clue what’s going to happen, but are we going to hold back positive for the thought of a negative?” (P07). Another concluded that he was already 70 years old, so he did not need to worry about genetic discrimination or being fired from his job. This common dismissal of the risks of research enrollment might relate to the general lack of understanding of genetics as highlighted in Theme 1.

Interestingly, the most common risk described by clinicians was not related to stumbling upon an affirmative genetic finding that patients might not want to know about, but quite the opposite—that of not understanding what an abnormal variant meant for their patients in the first place. This relates to an altogether different category of risk related to transgressions of professional duty. One clinician described precision medicine research as having to be “comfortable with that uncertainty” (C08). Another clinician bemoaned that scientific advancement regrettably may lead to recognition of missed diagnoses, if they “look back in 5 years, and you didn’t even know the germline mutation that was bad was a bad one then, right?...Even if you didn’t know it was bad, should you have told them that something could be there?” (C10).

## Discussion

This analysis uniquely matches the perspectives of patient-participants with their corresponding clinicians, offering insight into the influence of the doctor-patient relationship on precision oncology research enrollment and satisfaction. Our findings highlighted nuanced challenges with informed consent to precision medicine, uniquely perceived benefits of precision oncology, and relatively discounted risks related to genomic discovery.

One key component of our findings relates to ensuring that patients have the capacity to fully understand the research to which they are being asked to consent. Specifically, although many patient-participants stated that they understood the basics of the science, the background they cited was limited to popular media and fictionalized interpretations, indicating low true genomic health literacy (defined as “the capacity to obtain, process, understand, and use genomic information for health-related decision making” [11]). The relative lack of genomic health literacy among patient-participants raises concerns for the maintenance of their underlying autonomy throughout the enrollment process and beyond.

A component of this genomic health literacy important to the process of informed consent is understanding the limitations of genome sequencing, a competency that has been associated with high levels of education [12]. For example, there is still a lack of common understanding of the term “actionable,” and there are differences in understanding “between patients and clinicians, with patients expecting more personal benefits to come from actionable results” [13]. Actionability generally relates to recognition of a germline mutation with implications for relatives, as well as identifying clinically prognostic biomarkers and biological targets to be used in the patient’s treatment. In head and neck precision oncology both remain rather rare; thus, there are more nebulous outcomes than direct benefits of enrollment at this stage.

Of the patients that do experience decisional conflict when enrolling in genomic sequencing, this phenomenon is associated with lower health literacy and a lack of experience with prior genetic testing [14]. Unfortunately, disparities in baseline genomic knowledge often persist longitudinally, despite the offering of educational materials and genetic counseling opportunities [15]. In this study, clinicians noted several times that the inherent vulnerability of their patients to both structural and individual coercion, or at least undue influence, to enroll in research was tied closely to clinical caregiving. Past research has demonstrated that framing potential benefits as aspirational, direct, and collateral can help clarify the otherwise complex relationship between research and clinical care in this space [16,17]. Our findings are consistent with these, confirming the need for better strategies to educate and counsel patients and participants alike.

The benefits of obtaining high genomic health literacy are that greater baseline knowledge of genomics has been associated with lower levels of distress related to participating in a genome sequencing study and higher levels of understanding of the study. Ensuring that both clinicians and patient-participants understand the risks and benefits of research participation can serve to clarify decisions and better enable prospective participants to honor their autonomy.

Although informed consent has been shown to improve knowledge about both the limitations and benefits of genome sequencing in a variety of settings [4,12], many oncologists have little familiarity with newer genetic technologies and have a low level of genomic literacy themselves, as several of our clinician interviewees admitted [18]. Clinicians without backgrounds in genetics also report difficulty understanding and communicating genomic terminology and the volume of complex information yielded from genomic sequencing studies [19]. If clinicians have a limited understanding of genetic sequencing studies, they may be uncomfortable communicating the goals or results of these studies to their patients. This could lead to lower levels of physician satisfaction and less participation in future studies [18]. This tension was noted by the clinicians interviewed herein as well, despite the fact that they are all engaged in academic research in this field.

The theme of altruism is also prominent in studies exploring subjects’ motivations to engage in genetic research [20]. In the broadest lens, this reflects contributing to the generation of generalizable knowledge to help future patients—the cornerstone of clinical research itself. However, this concept is far more nuanced when considering the distinctions between germline and somatic mutations [21]. In this study, in which somatic mutations are far more common than germline mutations in a head and neck cancer cohort, the likelihood of family members benefitting directly from the research is lower. An intriguing ethical analysis reconceptualizes participation in precision medicine “as inextricable from social relationships and their ongoing ethical obligations. Going beyond altruism, reframing biospecimen and data collection in terms of socially regulated gift-giving recovers questions of responsibility and care…and underscores ethical commitments to reciprocity and responsibility” [22].

In summary, patient-participants and their providers offered complementary and nuanced perspectives on their motivation to engage in precision head and neck oncology research. It is important to note that the findings reported here represent the views of a specific group of clinicians and their patient-participants. Further research is warranted to generalize their experiences. Nevertheless, this study reflects the participants’ excitement to be a part of cutting-edge research, as well as their inherent altruistic tendencies. This enthusiasm should still be tempered with realistic expectations, and better systems should be created to educate cancer patients turned participants about the precision medicine.

## Abbreviations

C: clinician
MiOtoSeq: Michigan Otolaryngology and Translational Oncology Sequencing Center
P: patient
